# Eco-Friendly Solar-Powered H_2_ Generation from Plastic Waste Using Earth-Abundant Cu-Doped ZnS Catalysts

**DOI:** 10.3390/nano15171311

**Published:** 2025-08-26

**Authors:** Zhen Li, Ye Wang, Kwang Leong Choy

**Affiliations:** Suzhou Key Laboratory of Advanced Sustainable Materials and Technologies, The Environmental Research Center, Division of Natural and Applied Sciences, Duke Kunshan University, Kunshan 215316, China

**Keywords:** non-toxic catalyst, structure control, photoreforming plastic, H_2_ evolution

## Abstract

The photoreforming of plastics into fuel and small organic molecules at ambient temperature presents a sustainable alternative to landfills and incineration. However, most existing photocatalysts depend on noble or toxic metals, limiting their environmental compatibility. Here, we present a non-toxic, eco-friendly Cu-doped ZnS photocatalyst synthesized via a simple one-pot wet chemical method for efficient plastic waste conversion in an alkaline solution. This earth-abundant catalyst exhibits broad visible light absorption and exceptional charge transfer efficiency, enabling high photocatalytic activity. By optimizing Cu doping levels, we achieve a promising H_2_ generation rate of 201.5 μmol g^−1^ h^−1^. We elucidate the photoreforming mechanism, paving the way for scalable and sustainable plastic upcycling.

## 1. Introduction

Plastic, one of the most creative inventions, is widely used in daily life due to its durability, low cost, and facile synthesis. However, the thermal stability and chemical inertness of waste plastic make it difficult to decompose. Furthermore, with the continuous advancement of the application of plastics in various industries, large amounts of waste plastics have begun to accumulate in landfills or be discarded into the environment, posing serious harm to the global ecosystem [1,2]. Driven by global environmental protection policies, the exploration of eco-friendly and economical approaches for the sustainable recycling of plastic waste has become a research hotspot across various fields [3,4,5,6]. Compared with physical degradation methods (e.g., landfilling and incineration), chemical recycling techniques (such as pyrolysis, solvolysis, gasification, hydrogenation, and steam or catalytic cracking) [7] often require high temperatures and pressures, may use toxic catalysts, and may generate harmful emissions. Photoreforming technology presents a highly promising strategy for plastic valorization, attracting growing interest in both academic and industrial areas [8]. This technology emphasizes the direct use of solar energy to convert waste plastics into hydrogen gas and small-molecule organic compounds, from starting monomers to hydrocarbons. At the same time, these products can not only be repolymerized into plastics, but also upgraded into high-value-added products and fuels, thereby achieving the closed-loop material recycling system and reuse of materials [2,9]. Therefore, photocatalytic valorization offers a promising solar-to-fuel conversion approach to simultaneously address contemporary waste management and energy sustainability challenges [1,4,10].

The photocatalytic conversion of plastics was first reported by Kawai and Sakata in their seminal 1981 study, which demonstrated hydrogen production through photodecomposition of polyvinyl chloride (PVC) in aqueous media using a Pt/TiO_2_ catalyst [11]. Their study revealed that increasing the alkalinity of the reaction solution was beneficial to the production of hydrogen, which was due to the oxidizing power of radical •OH which could form easily from OH-. Nevertheless, the efficacy of this system is limited by its modest catalytic activity and its exclusive sensitivity to ultraviolet radiation. Reisner’s research group reported a green photoreforming technology of plastics at ambient temperature in 2018. They pointed out that CdS/CdO_x_ quantum dots exhibited photocatalytic hydrogen evolution performance for a variety of plastic polymers under alkaline conditions. Its hydrogen evolution performance during the photoreforming of polylactic acid (PLA) reached 64.3 ± 14.7 mmol g^−1^ h^−1^, while for polyethylene terephthalate (PET) it was only 3.42 ± 0.87 mmol g^−1^ h^−1^ [12]. This has been a big step forward in the sustainable recycling of plastics in ambient conditions. Meanwhile, CdS also demonstrates excellent catalytic performance in photocatalytic water splitting for hydrogen production. Wang et al. [13] successfully synthesized an efficient CdS@g-C_3_N_4_ photocatalyst that demonstrates remarkable hydrogen evolution activity through water splitting, achieving an impressive hydrogen production rate of 19.88 mmol g^−1^ h^−1^. Notably, the catalyst exhibits exceptional stability, maintaining its high hydrogen evolution performance even after multiple consecutive reaction cycles.

Compared with CdS, ZnS, as a low-cost, non-toxic semiconductor material with excellent charge transfer properties, demonstrates remarkable potential for photocatalytic applications [14]. However, its relatively wide band gap (~3.7 eV) limits visible light absorption efficiency, while severe photogenerated charge recombination further restricts its photocatalytic performance [14]. To enhance the photocatalytic activity of ZnS, various modification strategies have been developed. Particularly, metal ion doping has attracted considerable attention due to its dual functionality: serving as electron traps to suppress e^−^-h^+^ recombination and simultaneously modulating the band structure to induce absorption edge redshift [15]. Research confirms that copper doping exhibits particularly outstanding effects in improving the performance of sulfide-based photocatalysts. For instance, Xu et al. [14] fabricated a Cu-doped ZnS catalyst (denoted as 5CZ) via a facile one-step hydrothermal method, which demonstrated exceptional photocatalytic performance with a remarkable hydrogen evolution rate of 8737.9 μmol g^−1^ h^−1^ through water splitting. Li et al. [16] developed an innovative Cu-doped ZnS (Cu-ZnS) nanoarchitecture with a three-dimensional open framework through a well-designed two-step protocol combining anisotropic etching and subsequent sulfidation. The as-prepared 1%Cu-ZnS framework exhibits benchmark photocatalytic performance, delivering an exceptional solar-driven hydrogen evolution rate of 8.30 mmol h^−1^ g^−1^ without any cocatalysts, coupled with remarkable stability. This achievement represents one of the highest reported activities among state-of-the-art ZnS-based photocatalysts. While ZnS has demonstrated excellent photocatalytic performance for hydrogen production via water splitting, its application in photocatalytic PET reforming for hydrogen generation has not been reported to date.

**Figure 1:**
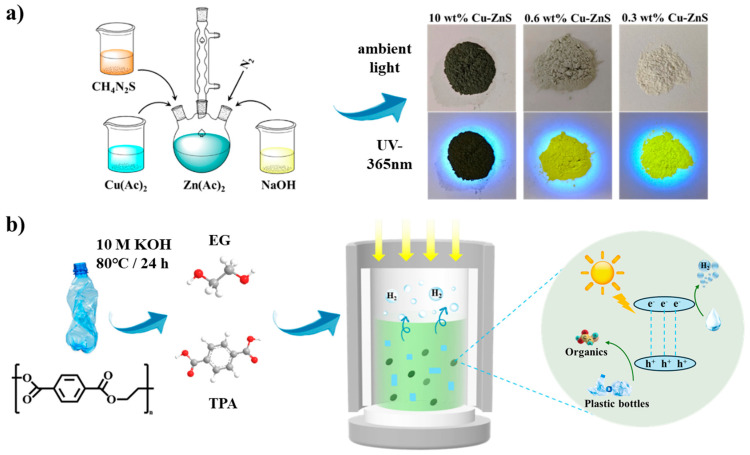
(**a**) Schematic diagram of Cu-doped ZnS synthesis and a color comparison of ZnS at different Cu doping concentrations under UV illumination. (**b**) Schematic diagram of the photoreforming process of waste plastic using Cu-doped ZnS catalysts.

Here, we report a facile one-pot synthesis of a non-toxic and earth-abundant catalyst based on Cu-doped ZnS. Compared with previous Cd-based catalysts and their complex synthesis steps, this catalyst is more eco-friendly, low-cost, and simple to synthesize. Meanwhile, the effects of different amounts of Cu doping on the morphology, structure, and properties of the Cu-doped ZnS catalysts were investigated. Interestingly, as the Cu content increases, the color of the synthesized sample is observed to gradually darken. Consequently, the visible light absorption range would increase, and the sample may be in the shape of nanoflowers. Therefore, the photocatalytic hydrogen evolution performance can be effectively improved by rationally regulating the amount of doping Cu in the ZnS to achieve structural optimization and interface control. The schematic diagram of the photoreforming process is shown in Figure 1b. Under alkaline conditions, polyethylene terephthalate (PET) undergoes hydrolysis, yielding ethylene glycol (EG) and terephthalic acid (TPA), which then adsorb onto the catalyst surface. Upon light irradiation, these adsorbed intermediates participate in simultaneous redox reactions: an oxidative pathway breaks them down into smaller organic molecules, while a reductive pathway generates clean hydrogen fuel (H_2_).

This work provides a new approach for the design of non-toxic, low-cost catalysts, and offers a new catalyst for the photoreforming of plastics.

## 2. Experimental Section

### 2.1. Materials

Zinc acetate (Zn(CH_3_COO)_2_), copper acetate (Cu(CH_3_COO)_2_), thiourea (CH_4_N_2_S), sodium hydroxide (NaOH), and absolute ethanol were used in the synthesis of Cu-doped ZnS catalysts. The above chemicals were all reagent grades and come from Admas (Shanghai, China). Polyethylene terephthalate (PET, granular) was obtained from Merck (Shanghai, China). All chemicals were of analytical reagent grade and used without further purification. The water used in all experiments was purified by a Millipore system form Merck (Shanghai, China).

### 2.2. Synthesis

Cu-doped ZnS was synthesized under mild conditions via a simple one-pot method. In general, a solution was prepared by dissolving a mixture of Cu(CH_3_COO)_2_ (0.02 M) and Zn(CH_3_COO)_2_ (0.5 M) in 80 mL of deionized water within a three-necked flask, and was stirred continuously for 5 min. Subsequently, 1.5 M of 40 mL thiourea was slowly added to the solution mixture and stirring was continued for an additional 5 min. A small quantity of NaOH was then introduced to the solution mixture to adjust the pH to 7. Finally, the total solution was heated to 100 °C and reacted for 6 h. Then, the obtained x wt% Cu-ZnS samples were separated from the reaction solution by washing with H_2_O and ethanol, then centrifuging at 10,000 rpm. The resultant 0.6 wt% Cu-ZnS was dried at 60 °C overnight. The Cu doping amount was varied accordingly to obtain 0.3 wt% Cu-ZnS and 10 wt% Cu-ZnS.

### 2.3. Characterization

X-ray powder diffraction (XRD) spectra were performed on a benchtop Aeris equipped with Cu Kα radiation with the scanned range of 10–80°. Scanning electron microscope (SEM) images were obtained using an S4800. High-resolution transmission electron microscope (HRTEM) images and energy-dispersive X-ray spectroscopy (EDX) were conducted on a Fei G2 F30 to examine morphologies and elemental compositions. Raman Spectra were recorded on a HORIBA LabRAM HRE Evolution with a 325 nm laser as an excitation source. Electron paramagnetic resonance (EPR) was measured on a Bruker-E500. The visible absorption range and band gap were recorded by UV–vis diffuse reflectance spectroscopy (DRS) (Shimadzu, Kyoto, Japan). X-ray photoelectron spectroscopy (XPS) was recorded on a Thermo Fisher ESCALAB XI + (Thermo Fisher Scientific, Waltham, MA, USA). UV–vis diffuse reflectance spectroscopy (UV-DRS) spectra were tested on a Shimadzu UV-2600i. Transient photoluminescence (PL) spectra were obtained at room temperature using a FLS-1000 spectrometer. Steady PL spectra were determined with an RF-5301PC Spectro fluorophotometer (Shimadzu). Inductively coupled plasma optical emission spectrometry (ICP-MS) measurements were carried out using an Agilent 720 (Agilent Technologies, Santa Clara, CA, USA). ^1^H NMR was reformed using a Bruke 400M to obtain the structural composition of the photocatalytic compounds.

### 2.4. Pre-Treatment of Plastic Substrate

The 4 g of PET powder was dispersed in 80 mL of the 10 M KOH solution and placed in a three-neck bottle. The solution was heated at 80 °C for 24 h. After the reaction was completed, the solution was cooled to room temperature and centrifuged to remove the supernatant for the subsequent photocatalytic test. The PET bottle was sheared into small-size pieces and treated as described above where the concentration of the substrate was subjected to the identical pre-treatment.

### 2.5. Photocatalytic Performance Evaluation

In the photoreforming test conducted under ambient conditions, 20 mg of the Cu-ZnS photocatalyst was dispersed in 50 mL of 25 mg/mL pre-treated PET supernatant. The reactor was purged with high purity N_2_ for 10 min to remove any residual air. A 300W Xenon lamp (PLS-SXE 300, Perfectlight, Beijing, China) was used as the light source that was equipped with a UV-cut filter with a 400 nm cutoff wavelength to provide visible light irradiation. H_2_ generation was monitored periodically by analyzing samples taken from the reactor head space gas using gas chromatography (Fuli).

In the photoreforming test in vacuum, the reactions were performed in a quartz glass vessel connected to an All Glass Automatic On-line Trace Gas Analysis System (Labsolar-6A, Perfectlight, Beijing, China). Typically, 20 mg of 0.6 wt% Cu-ZnS was added into 50 mL of 5 M KOH solution under stirring. The reaction system was first evacuated to remove air and then injected with 20 mL of Ar. A 300 W xenon lamp (PLS-SXE 300, Perfectlight, Beijing, China) with a UV-cutoff filter (λ > 400 nm, light intensity: 1.5 W cm^−1^) was used as the light source while the reaction temperature was maintained at 25 °C using a low-temperature thermostat tank. The evolved H_2_ was continuously monitored and analyzed by gas chromatography (Fuli) with Ar as the carrier gas.

### 2.6. Photoelectrochemical Test

Photoelectrochemical measurements of the samples were analyzed with an electrochemical workstation (CHI 760E) using a conventional three-electrode system. The Pt electrode and Ag/AgCl electrode (saturated with KCl) were used as the counter electrode and reference electrode, respectively. The working electrode was prepared on an indium tin oxide (ITO) glass plate (10 × 20 mm) according to the following method: 6 mg of the sample was dispersed in a solution containing 700 µL of H_2_O and 200 µL of C_2_H_5_OH, which was sonicated for 30 min and then dropped uniformly onto a glass coated with indium tin oxide (ITO) with a working area circa 0.2 cm^2^. Transient photocurrent responses, electrochemical impedance (EIS), the cyclic voltammetry (CV) curve, and the Mott–Schottky plot were performed in an electrolyte of 0.2 M sodium sulfate.

## 3. Result and Discussion

### 3.1. Synthesis, Composition, and Structural Analysis

Different amounts of Cu-doped ZnS (x wt% Cu-ZnS) catalysts were synthesized using a simple one-pot method (Figure 1a). The desired sample was obtained by simply using copper acetate, zinc acetate, and thiourea as precursors and refluxing at 100 °C. Here, the low Cu dopant (0.3 wt% and 0.6 wt%) would have a significant impact on the color of the sample, thereby affecting the fluorescence intensity of the sample. A 10 wt% Cu-doped photocatalyst was used as a typical reference for comparison in this investigation [17]. Inductively coupled plasma (ICP-MS) was used to quantify the composition of the samples. The mass ratios of Cu, Zn, and S in different Cu dopings in ZnS are summarized in Table S1, and they are consistent with the EDX results.

**Figure 2:**
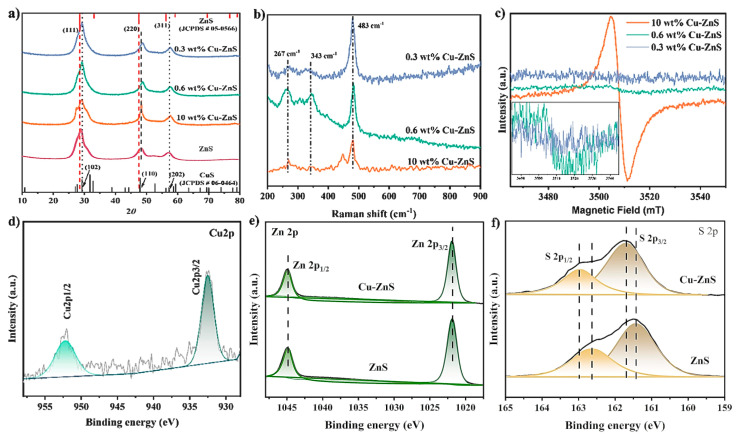
(**a**) The XRD patterns, (**b**) Raman spectra, and (**c**) EPR spectra of xCu-ZnS, the inset shows the magnified EPR patterns of 0.3 wt% and 0.6 wt% Cu-ZnS in the range of 3485–3545 mT. High resolution spectra of pristine ZnS and 0.6 wt% Cu-ZnS: (**d**) Cu 2p; (**e**) Zn 2p; and (**f**) S 2p.

The crystal structure of the synthesized samples was revealed by X-ray diffraction (XRD) patterns [18]. The Cu-doped ZnS showed a cubic crystal structure as shown in Figure 2a. Three broad peaks in the XRD pattern at circa 2θ = 9.2°, 48.5°, and 57.6° revealed the formation of a pure-phase polycrystalline Cu-ZnS with cubic zinc blende structure (β-ZnS phase) and good crystalline quality [19,20,21]. Comparing the PDF cards of ZnS (JCPDS, card no. 05-0566) and CuS (JCPDS, card no. 06-0464), the peaks exhibited a slight shift toward higher angles, which is probably caused by the doping of Cu^2+^ [19,22]. Since the ionic radius of Zn^2+^ (3.1 Å) is fairly similar to that of Cu^2+^ (3.2 Å), there is a high probability for the incorporation of Cu atoms into the ZnS lattice [23]. Further analysis of the (220) plane in Figure 2a reveals that the samples with Cu doping concentrations of 0.3 wt% and 0.6 wt% show slight peak shifts, potentially due to lattice distortion from Cu incorporation [24]. However, at higher doping levels, the (220) diffraction peak of the 10 wt% Cu-ZnS sample becomes significantly sharper. This sharpening was likely caused by the formation of a secondary CuS phase due to excessive doping, as confirmed by its alignment with the (110) plane of CuS [17].

Furthermore, Raman spectroscopy can provide additional information regarding the phase identification and crystalline nature of the obtained sample [23]. As shown in Figure 2b, three distinct peaks have been observed, with a strong vibrational mode at 476 cm^−1^, which could be assigned to the xCu-ZnS as E2 (high) mode [25,26], whereas the peak at 476 cm^−1^ could be assigned to S−S stretching in xCu-ZnS [27]. Raman peaks appeared at circa 267 cm^−1^ and 343 cm^−1^, which could be derived from ZnS [28]. Apart from the right shift, the peak intensity tends to reduce with an increase in the Cu dopant, which might be attributed to the dopant of Cu inducing lattice phase transitions and defects [22,23,25]. In addition, we also observed a peak at 448 cm^−1^ in the 10 wt% Cu-ZnS, which might be the peak of CuS, and this peak disappeared as the Cu content decreased. Additionally, electron paramagnetic resonance (EPR) spectra provided fingerprint evidence for probing the copper entities, and Figure 2c shows a sharp signal at 3210 mT, which is attributed to copper entities [29]. Obviously, the 10 wt% Cu-ZnS (reference sample) shows the strongest EPR signal, which might be due to the presence of more unpaired electrons, enhancing delocalization. Therefore, the relative SERS intensity in the resulting Cu-ZnS photocatalyst could be adjusted by varying the content of the Cu precursor. With the decrease in Cu content, the signal at 3210 mT is obviously weakened. This again proves that Cu has been successfully incorporated into the ZnS lattice.

The surface elemental composition of the 0.6 wt% Cu-ZnS has been estimated using X-ray photoelectron spectroscopy (XPS) as shown in Figure 2d–f [30]. The obtained spectra are corrected on the C1s peak at 284.5 eV. Compared to pristine ZnS, the XPS survey spectra of Cu-doped ZnS (Figure 2d–f) exhibit additional peaks corresponding to elemental copper. Peaks from Zn and S are observed in both spectra. The binding energies of Zn 2p3/2 and Zn 2p1/2 are located at 1022.15 eV and 1045.06 eV, respectively, consistent with Zn (II) [31]. The binding energies of S 2p3/2 and S 2p1/2 are identified at 161.56 eV and 162.73 eV, matching the reported values for sulfur in ZnS [31]. Notably, the peak positions of both Zn and S shift toward higher binding energies, suggesting an interaction between ZnS and Cu [31]. However, the Cu 2p3/2 and Cu 2p1/2 peaks appear at 932.08 eV and 951.98 eV. These peaks can be assigned to either metallic Cu^0^ or cationic Cu^+^ species, as the characteristic satellite peaks of Cu^2+^ (942 eV and 963 eV) were not detected [31,32]. As shown in Figure S1, the presence of small-intensity peaks corresponding to C1s and O1s noted in the survey spectra might be due to atmospheric contamination over the surface, and no other elements have been detected. Hence, it can be concluded that the synthesized catalyst is composed of Cu, Zn, and S without any other elemental impurities.

**Figure 3:**
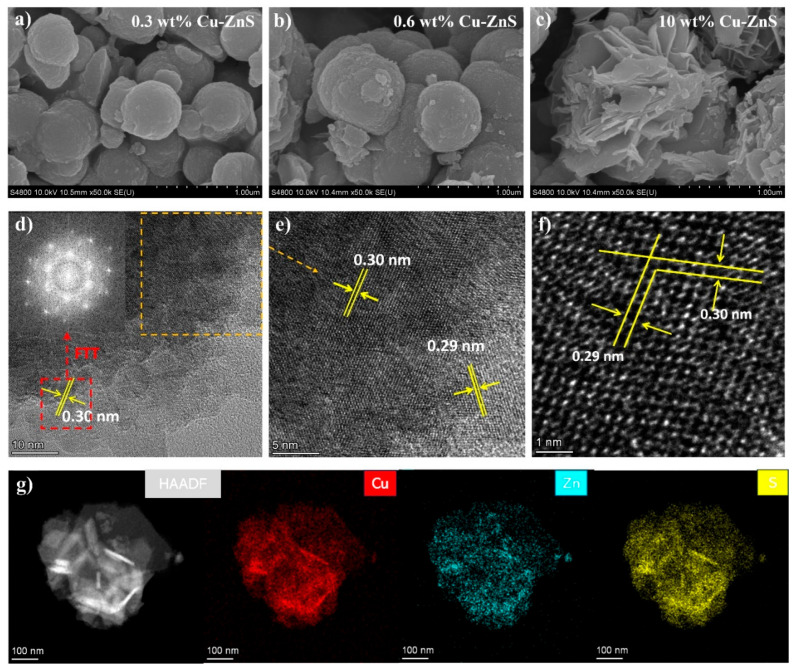
(**a**–**c**) SEM images of 0.3 wt% Cu-ZnS, 0.6 wt% Cu-ZnS, and 10 wt% Cu-ZnS. (**d**–**f**) Different magnification TEM images and SAED pattern of 0.6 wt% Cu-ZnS. (**g**) High-angle annular dark field (HAADF) image and the corresponding EDX mappings of 0.6 wt% Cu-ZnS.

Scanning electron microscopy (SEM) and transmission electron microscopy (TEM) were used to analyze the surface morphology and structure of the catalytic samples: 0.3 wt% Cu-ZnS; 0.6 wt% Cu-ZnS; and 10 wt% Cu-ZnS, as shown in Figure 3. Interestingly, it can be observed that the proportion of Cu significantly influences the microstructure of the Cu-ZnS sample (Figure 3a–c and Figure S2). At a high Cu content, the samples would consist of small petal-like structures with nano-sized dimensions and uniform distribution [18,23,33]. However, as the Cu content is reduced, the sample structure tends to become more spherical, with fine nano-sized particles appearing on the surfaces of the spheres. The microstructure and elemental composition of the samples were further investigated by TEM [34]. As can be seen in Figure 3d–f, 0.6 wt% Cu-ZnS shows the lattice fringes with spacing of 0.30 nm, which corresponds to the ZnS (111) (Figure S3). Due to the lattice phase transfer and defects caused by the incorporation of Cu ions, the lattice spacing is reduced to 0.29 nm, which corresponds to the Raman results. Elemental EDX mapping performed on the area depicted in the high-angle annular dark field (HAADF) image shows that Cu, Zn, and S are uniformly distributed throughout the sample (Figure 3g), further demonstrating the successful incorporation of Cu into ZnS. Simultaneously, the EDX spectra clearly show the signals of Cu, Zn, and S, and their corresponding proportions (Figure S4).

### 3.2. Opto-Electrochemical Performance

**Figure 4:**
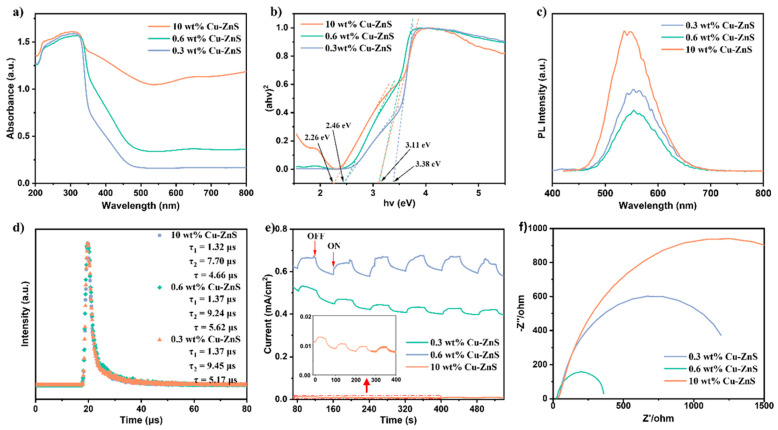
(**a**) The UV–vis absorption spectra, (**b**) the band gap energy estimations based on Kubelka–Munk plots of Cu-ZnS, (**c**) the fluorescence intensity, (**d**) time-resolved photoluminescence spectra, (**e**) photocurrent response, and (**f**) EIS Nyquist plots for xCu-ZnS.

The characterization of the optical properties and electronic energy band structures of the Cu-ZnS samples was carried out using the UV–vis diffuse reflectance spectrum (UV-DRS) [35,36]. As shown in Figure 4a,b, the visible light absorption range has significantly expanded with the increase in the Cu dopant, accompanied by the darkened color. Furthermore, Cu doping would introduce impurity energy levels that can change the energy band structure and optical absorbance of the sample by providing additional electronic states within the band gap. As can be seen from Figure 4b, the synthesized catalysts exhibited band gap energies in two ranges: 3.1–3.4 eV and 2.2–2.5 eV, respectively, which shows excellent agreement with the reported band gap of cubic-phase nanoparticles [37]. Simultaneously, the dual band gap in Figure 4b confirmed the alloy nature of the material. The higher band gap can transmit high-energy photons that fall on the material, while the lower band gap facilitates light energy conversion [27]. Additionally, given that the impurity energy levels arise within the band gaps of photocatalysts, the introduction of metal dopants could mitigate electron–hole recombination, enhancing the transport of charge carriers [28,38]. The energy band structure of n-type semiconductor-based catalysts was investigated by Mott–Schottky in Figure S8. The flat band potentials of 0.3 wt% Cu-ZnS, 0.6 wt% Cu-ZnS, and 10 wt% Cu-ZnS were estimated to be −0.65 V, −0.62 V, and −0.87 V (vs. RHE), respectively. Correspondingly, the conduction band (CB) potential of these x wt% Cu-ZnS catalysts are −0.85 eV, −0.82 eV, and −1.07 eV. The valence band (VB) potential was calculated using the DRS data and the equation EVB = Eg + ECB [36], resulting in an energy band diagram (Figure S8). (ECB, Eg, and EVB are the conduction band (CB) potential, sample band gap, and valence band (VB) potential, respectively.)

**Figure 5:**
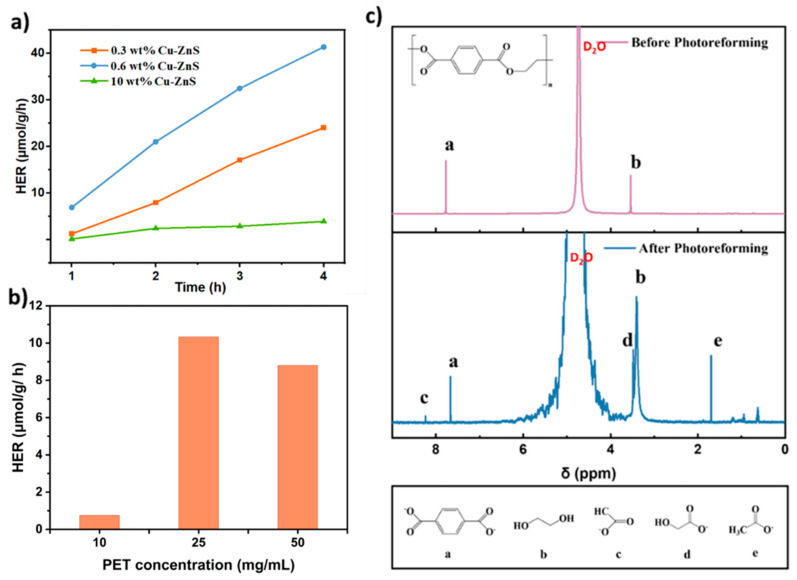
(**a**) The time-dependent evolution of H_2_ with different Cu contents on photocatalytic performance. (**b**) The effect of different KOH concentrations on photocatalytic performance. (**c**) The ^1^H NMR spectra for substrates of PET following photoreforming (a: Terephthalate (TPA); b: Ethylene glycol (EG); c: Formate; d: Glycolate; e: Acetate.)

To further explore the separation, migration, and recombination of photogenerated charge carriers, the steady-state photoluminescence (PL) was studied [39,40]. As shown in Figure 4c, the fluorescence intensity of 0.6 wt% Cu-ZnS is the weakest as compared to 0.3 wt% Cu-ZnS and 10 wt% Cu-ZnS, which means that this catalyst is more effective in accelerating the separation/transfer of electron–hole pairs. In addition, time-resolved PL spectroscopy was used to explore the lifetime of the carriers (Figure 4d). Interestingly, the fluorescence lifetime of 0.6 wt% Cu-ZnS (5.62 μs) is much higher than those of 10 wt% Cu-ZnS (4.66 μs) and 0.3 wt% Cu-ZnS (5.17 μs), which would help for the migration of photogenerated charge carriers in the photocatalytic reactions; this is consistent with the photocatalytic test results (Figure 5a). The lifetime of excited states is intrinsically correlated with defect states [41]. Copper doping introduces copper vacancies into the system, where the doping sites exhibit dual functionality: (i) serving as charge carrier traps that promote charge separation and enhance photocatalytic activity, and (ii) simultaneously acting as crystalline defects that function as recombination centers for photogenerated electron–hole pairs. Notably, higher doping concentrations lead to increased defect formation. When defect concentration exceeds the optimal level, it significantly shortens excited-state lifetime and accelerates electron–hole recombination through non-radiative pathways [42].

Generally, the activity of the photocatalyst is strongly related to the transfer and separation of photogenerated charge carriers. The transient photocurrent time curves of three catalysts are achieved by the on–off cycle detections of the electrodes under the visible light irradiation (Figure 4e) [43,44]. As evidenced by the EIS Nyquist plots (Figure 4f), the 0.6 wt% Cu-ZnS sample exhibits superior charge separation and transfer capabilities compared to the other photocatalysts. Figure 4f shows that the three photocatalysts display similar semicircles in high frequency. Among them, 0.6 wt% Cu-ZnS has the smallest radius, revealing the smallest interfacial charge transfer resistance and the fastest electron transfer ability [45,46]. According to the above analysis, the introduction of the appropriate Cu doping in ZnS can effectively promote the separation and transfer of photogenerated electrons and holes, which is consistent with the photocatalytic activity. The catalyst activity of the Cu-ZnS was determined by CV curves; the electrochemical active surface area (ESCA) was further obtained in order to analyze the HER activity (Figures S5–S7).

### 3.3. Photocatalytic H_2_ Evolution Coupled with Plastics Reforming

The photoreforming performance of the as-prepared x wt% Cu-ZnS samples in PET solution (25 mg/mL) from the commercial PET granules was conducted in a 100 mL stainless steel container. Generally, in order to destroy the durability and inertness of plastics, PET particles should be pre-treated in a KOH solution at 80 °C for 24 h to depolymerize them into corresponding monomer molecules [2,47,48]. Subsequently, the supernatant of the PET-depolymerized solution was collected and filtered using a filter with a pore-size of 0.22 μm, which could then be used as a starting material to drive the photoreforming reaction. The H_2_ evolution kinetics was periodically examined by gas chromatography over the x wt% Cu-ZnS photocatalyst. Initially, it could be observed that photocatalysts with different x wt% of Cu dopings have significant differences in H_2_ release activities. As shown in Figure 5a, 0.6 wt% Cu-ZnS exhibited an H_2_ release rate in 5 M KOH, reaching 41.33 μmol/g within 4 h at 40 °C, which was a promising non-toxic, low-cost, and earth-abundant catalyst for the photoreforming H_2_ production of plastic waste. Moreover, it can be observed that for a typical 10 wt% Cu-ZnS (reference sample), the photocatalytic activity would deteriorate. This decline might be attributed to excessive Cu doping in ZnS, which would accelerate the recombination of photogenerated electrons and holes. This is consistent with the measured photophysical properties. Moreover, these results are also consistent with the fluorescence lifetime measurements, where excessive Cu doping leads to defect aggregation that accelerates electron–hole recombination. On the contrary, if the amount of Cu doping has been reduced, the activity of the catalyst would also be reduced. Therefore, an appropriate amount of Cu is of great significance for improving the photocatalytic hydrogen production activity. Simultaneously, PET produces a variety of oxidation products that are comparable to those found in commercial plastics, as shown in the ^1^H NMR spectra in Figure 5c [49]. Overoxidized products were adetected and primarily existed as carbonate in the reaction solution. The pre-treated PET contains ethylene glycol (EG), terephthalate (TPA), and other complex small molecules. Thus, ethylene glycol can act as an electron donor to be oxidized in the PET photoreforming. The organic small molecules after the photoreforming of PET would include acetate, formate, and glycolate, as shown in Figure 5c [36].

To reduce the cost and corrosiveness of the system, the following experiments were conducted in 5 M KOH. As shown in Figure 5b, the concentration of PET also has a certain impact on the photocatalytic hydrogen evolution performance. As the concentration of PET increases, the amount of H_2_ evolution decreases, which could probably be due to the active sites of the catalyst being blocked by the insoluble components of PET, thus inhibiting the activity of the photocatalyst [50]. The stability of the photocatalyst was investigated by photocatalytic cycle experiments (Figure S9a). After four cycles, the catalyst still maintained about 74% catalytic activity, indicating that the catalyst has a certain stability. Furthermore, the used Cu-ZnS was then collected and characterized by XRD. The XRD pattern shown in Figure S9b, which does not show obvious change, demonstrates the stable crystal structure of Cu-ZnS after the photocatalytic reaction.

To enhance efficiency, the reaction was also conducted in vacuum using the Perfectlight 6A system. The reaction achieved a cumulative H_2_ production of 806 μmol g^−1^ over 4 h, with an average rate of 201.5 μmol g^−1^ h^−1^ (Figure S10b), which was higher than the test performed in the earlier analysis using the typical photoreactor operated in ambient conditions (Figure S10a). Under vacuum conditions, the reduced gas pressure significantly minimized the molecular collisions and interference, thereby facilitating efficient gas adsorption and desorption on the catalyst surface. This process is critical for photocatalytic hydrogen evolution: when water molecules adsorb onto the photocatalyst, they effectively capture photogenerated electrons from the conduction band, promoting the separation of electron–hole pairs and enhancing hydrogen production efficiency [51]. Conversely, excessively slow desorption rates may lead to active site blockage, ultimately impairing photocatalytic performance [51]. Thus, Cu-doped ZnS is a promising, non-toxic, low-cost, and earth-abundant catalyst for the photoreforming of plastic waste into hydrogen, especially when compared to the reported catalysts in Table S2.

## 4. Conclusions

In summary, a series of earth-abundant, eco-friendly, and non-toxic Cu-doped ZnS catalysts were successfully synthesized via a facile one-pot wet chemical method, demonstrating efficient solar-driven H_2_ generation coupled with plastic waste valorization. Under simulated solar irradiation, the optimized 0.6 wt% Cu-ZnS achieved an exceptional H_2_ evolution rate of 201.5 μmol g^−1^ h^−1^. The incorporation of Cu dopants not only extended the visible light absorption range but also facilitated charge carrier separation and transfer, as evidenced by photoelectrochemical and spectroscopic analyses. Notably, the catalyst retained over 74% of its activity after four cycles, highlighting its robust stability for practical applications. This work advances the design of non-toxic, low-cost, solar-powered photocatalysts by elucidating the dual role of Cu doping in ZnS: (i) enhancing visible light harvesting and (ii) optimizing interfacial charge dynamics for simultaneous plastic degradation and H_2_ production. The findings align with global sustainability goals, offering a green pathway to convert plastic waste into clean fuel (H_2_) using sunlight as the sole energy input.

## Data Availability

Data is contained within the article and Supplementary Material.

## Supplementary Materials

The following supporting information can be downloaded at: https://www.mdpi.com/article/10.3390/nano15171311/s1, Figure S1: XPS spectra of 0.6 wt% Cu-ZnS; Figure S2: SEM images of 0.3 wt% Cu-ZnS, 0.6 wt% Cu-ZnS, and 10 wt% Cu-ZnS at different magnifications; Figure S3: HATEM images and SAED images of 0.6 wt% Cu-ZnS.; Figure S4: (a) CV curves at different scan rates; and (b) Cdl values estimation for 0.3 wt% Cu-ZnS; Figure S5: (a) CV curves at different scan rates; and (b) Cdl values estimation for 0.6 wt% Cu-ZnS; Figure S6: (a) CV curves at different scan rate; and (b) Cdl values estimation for 10 wt% Cu-ZnS; Figure S7: Mott–Schottky plots of (a) 0.3 wt% Cu-ZnS; (b) 0.6 wt% Cu-ZnS; and (c) 10 wt% Cu-ZnS; Figure S8: Schematic diagram of energy band structures of x wt% Cu-ZnS; Figure S9: (a) Four consecutive photoreforming cycles of PET for H_2_; production using 0.6 wt% Cu-ZnS, and (b) XRD pattern comparison of the catalyst before and after reaction; Figure S10: (a) Hydrogen evolution rates over 5 h with varying Cu doping concentrations in ZnS; (b) HER performance of 0.6 wt% Cu-ZnS measured using a Perfectlight 6A; Table S1: ICP-MS and EDS elemental analysis of Cu-doped ZnS samples with varying doping ratios; Table S2: Comparison of plastic photoreforming performance of different photocatalysts [52,53,54,55,56].
