# Supplementary material for: Eco-Friendly Solar-Powered H2 Generation from Plastic Waste Using Earth-Abundant Cu-Doped ZnS Catalysts
Source: Nanomaterials (Basel). 2025 Aug 26;15(17):1311. doi: 10.3390/nano15171311 (PMC12430214; doi:10.3390/nano15171311)
Supplement: Supplementary file 1 [file nanomaterials-15-01311-s001.zip › nanomaterials-3823626-supplementary.pdf]

Supplementary information

Eco -Friendly Solar-Powered H_2 Generation from Plastic Waste Using Earth-Abundant Cu-Doped ZnS Catalysts

Zhen Li, Ye Wang and Kwang Leong Choy *

Suzhou Key Laboratory of Advanced Sustainable Materials and Technologies, The Environmental Research Center, Division of Natural and Applied Sciences, Duke Kunshan University, Kunshan 215316, China;
* Correspondence: kwang.choy@duke.edu

Table S1. ICP-MS and EDS elemental analysis of Cu-doped ZnS samples with varying doping ratios.

Weight (wt%)	ICP			EDX		
	Cu	Zn	S	Cu	Zn	S
10 wt% Cu-ZnS	11.1326	73.0996	15.7678	9.87	64.13	26
0.6 wt% Cu-ZnS	0.5568	78.0209	21.4223	0.77	76.85	22.38
0.3 wt% Cu-ZnS	0.2322	76.9698	22.7980	0.33	76.26	23.41

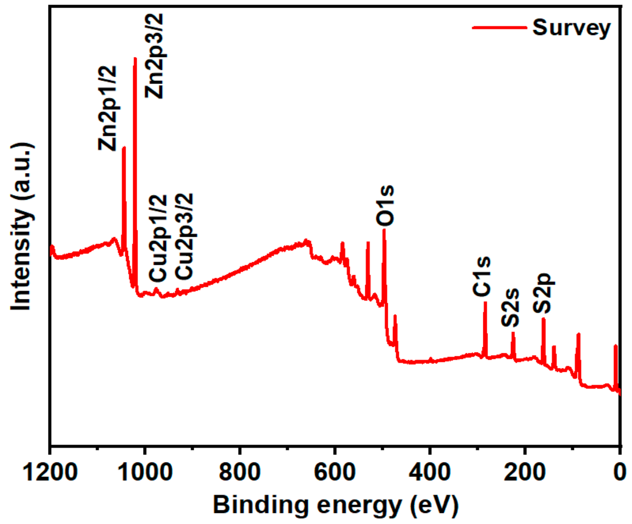

Figure S1. XPS spectra of 0.6 wt% Cu-ZnS.

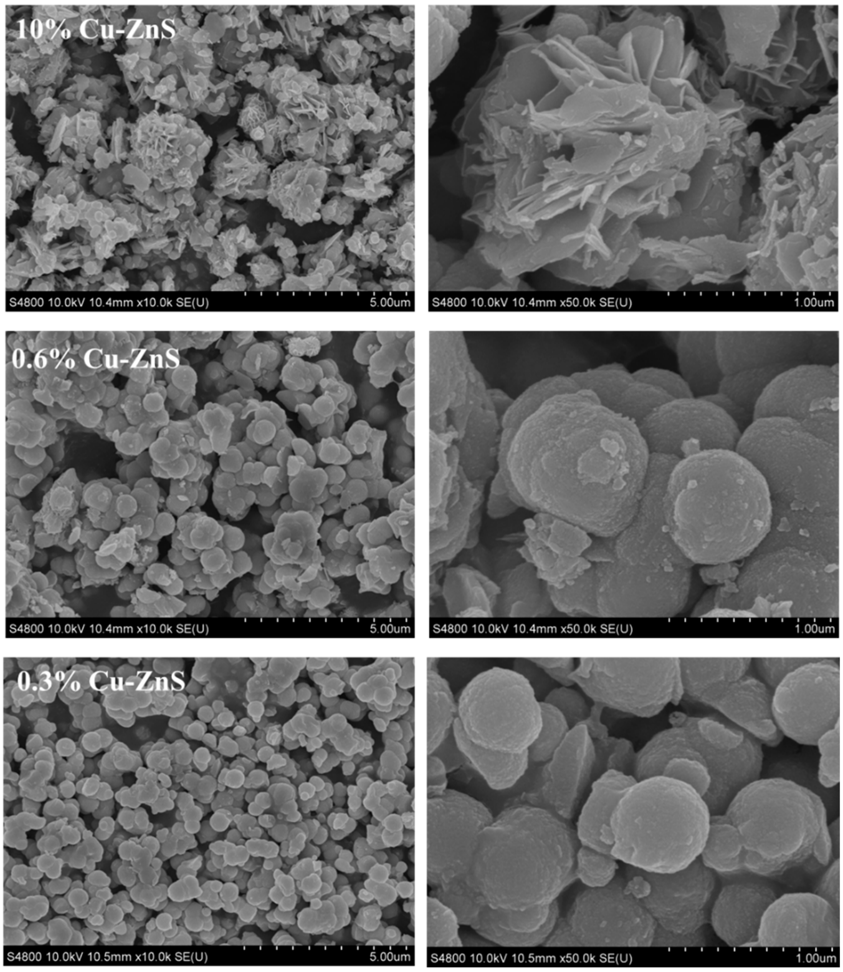

Figure S2. SEM images of 0.3 wt% Cu-ZnS, 0.6 wt% Cu-ZnS and 10 wt% Cu-ZnS at different magnifications.

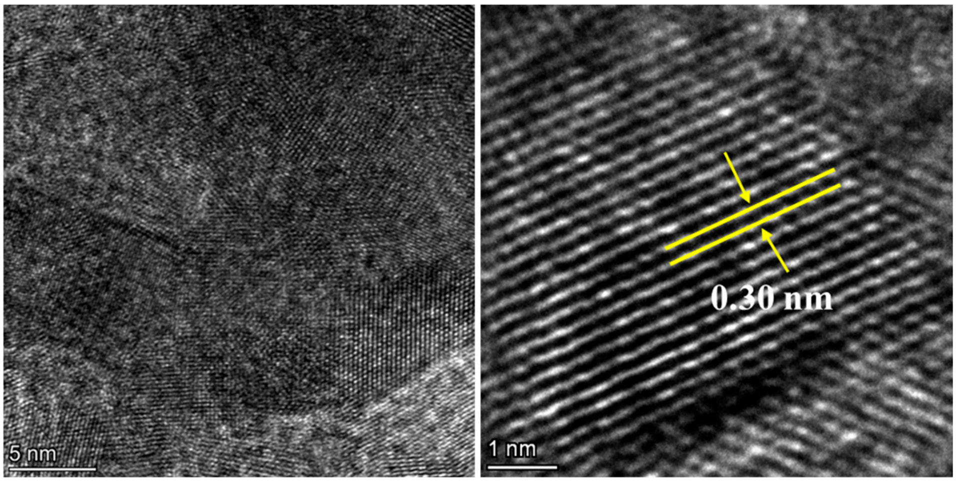

Figure S3. HATEM images and SAED images of 0.6 wt% Cu-ZnS.

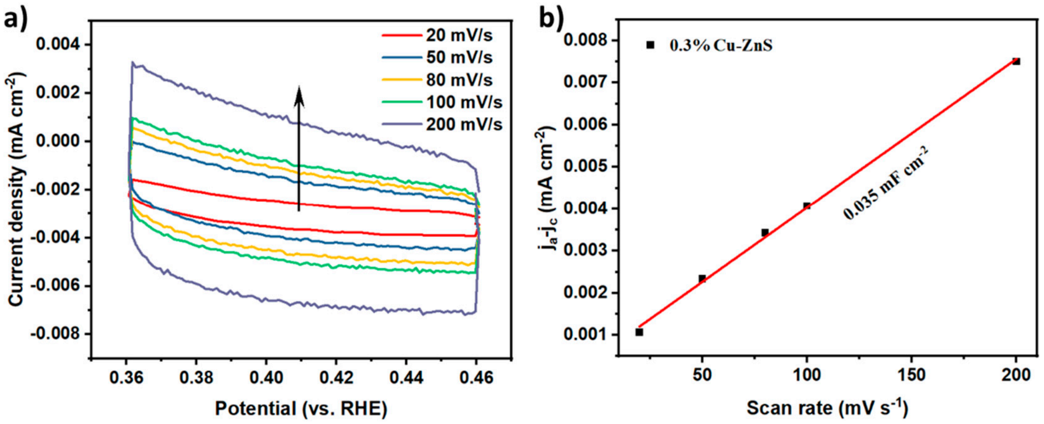

Figure S4. (**a**) CV curves at different scan rates; and (**b**) Cdl values estimation for 0.3 wt% Cu-ZnS**.**

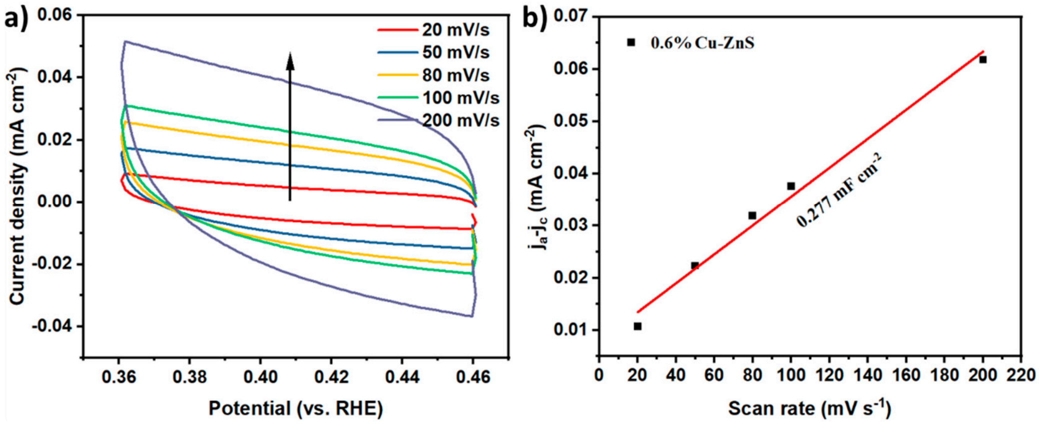

Figure S5. (**a**) CV curves at different scan rates; and (**b**) Cdl values estimation for 0.6 wt% Cu-ZnS.

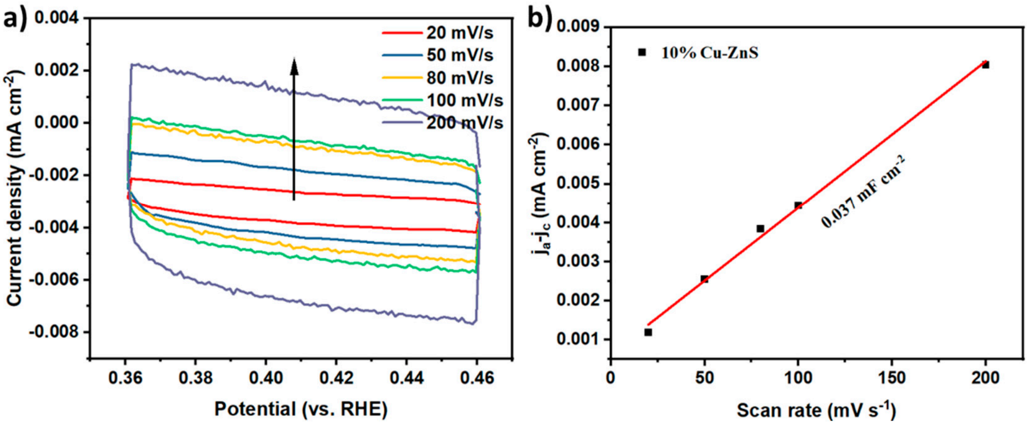

Figure S6. (**a**) CV curves at different scan rate; and (**b**) Cdl values estimation for 10 wt% Cu-ZnS.

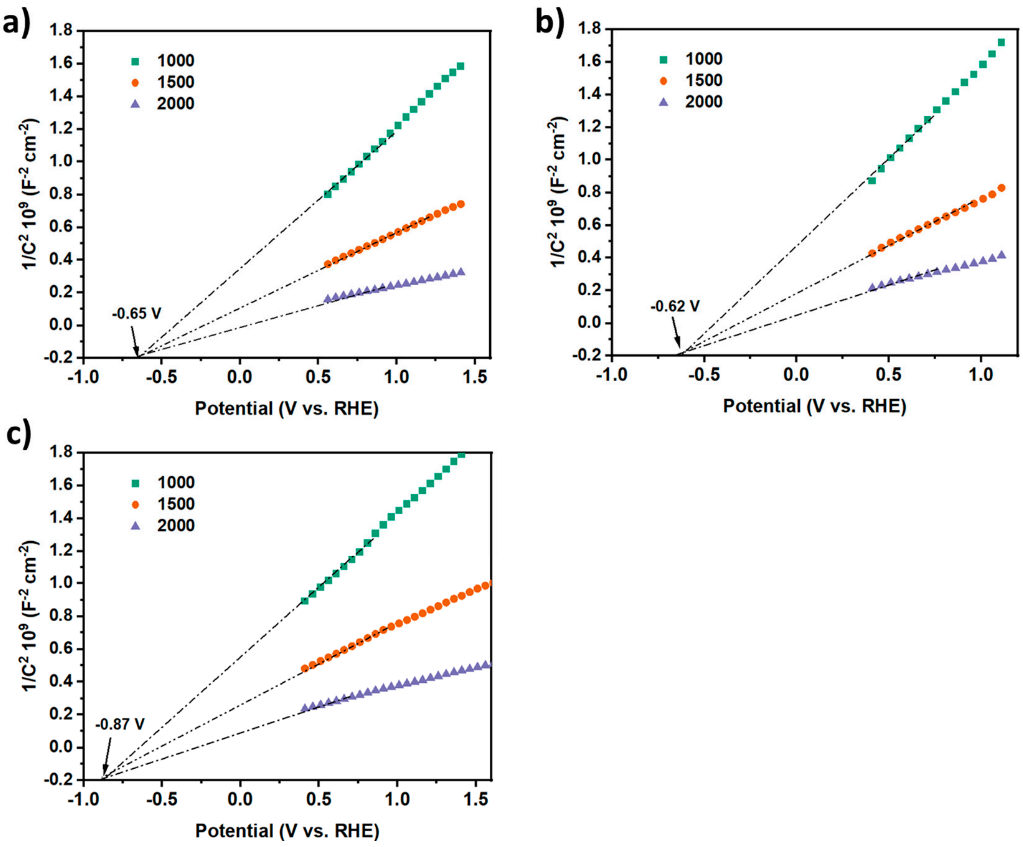

Figure S7. Mott Schoktty plots of (**a**) 0.3 wt% Cu-ZnS; (**b**) 0.6 wt% Cu-ZnS; and (**c**) 10 wt% Cu-ZnS.

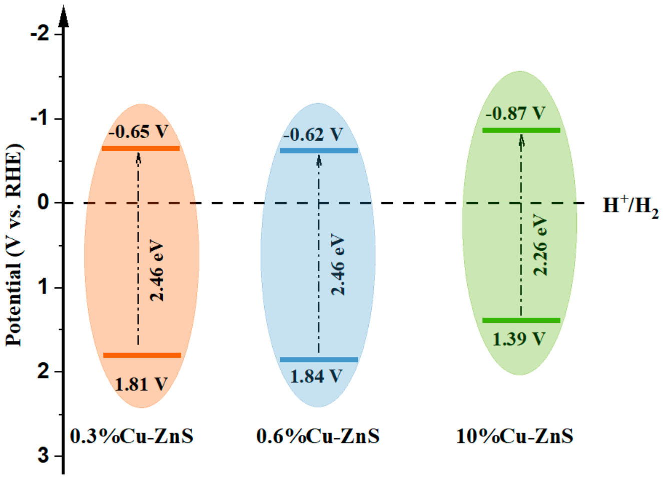

Figure S8. Schematic diagram of energy band structures of x wt% Cu-ZnS.

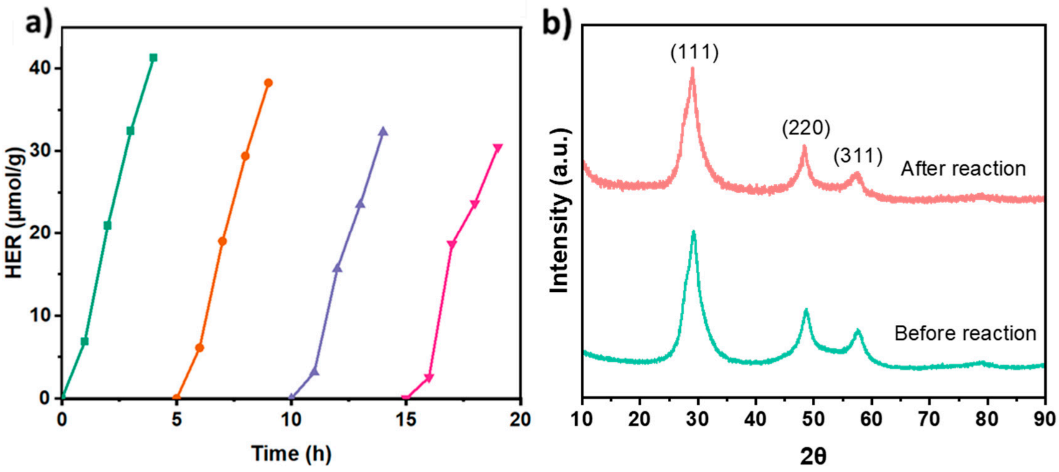

Figure S9. (**a**) Four consecutive photoreforming cycles of PET for H_2 production using 0.6 wt% Cu-ZnS, and (**b**) XRD pattern comparison of the catalyst before and after reaction.

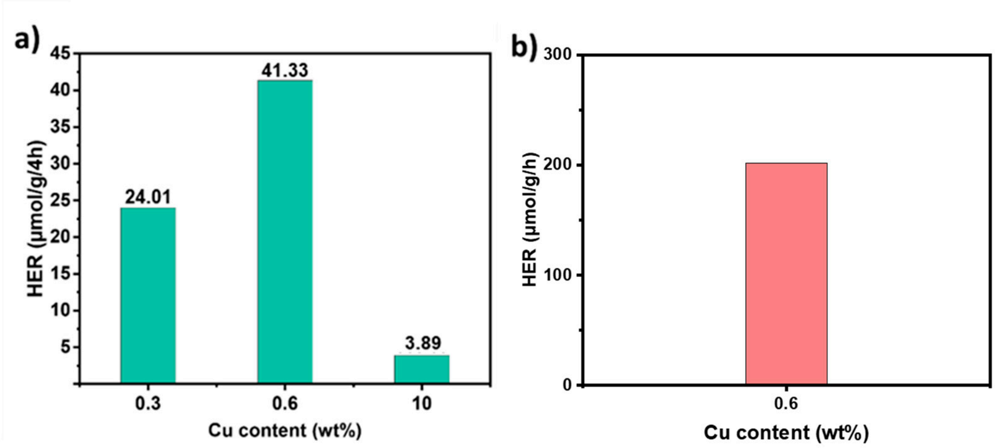

Figure S10. (**a**) Hydrogen evolution rates over 5 hours with varying Cu doping concentrations in ZnS; (**b**) HER performance of 0.6 wt% Cu-ZnS measured using a Perfectlight 6A

Table S2. Comparison of plastic photoreforming performance of different photocatalysts.

Photocatalyst	Substrate	Light source	Temperature	Catalyst amount	HER	Ref.
d-$NiPS_3$/CdS	50 mg/mL PET in 2 M KOH<br>50 mg/mL PET in 10 M KOH	300 W Xe lamp (λ > 400 nm)	Room temperature	1 mg	31380 μmol/g/h<br>121040 μmol/g/h	[1]
CdS/CdO_x	25 mg/mL PET in 10 M KOH	300 W Xe lamp (AM 1.5G)	25℃	1 nmol	3420 μmol/g/h	[2]
MXene/$Zn_{0.6}Cd_{0.4}S$	25 mg/mL PET in 10 M NaOH	300 W Xe lamp (λ > 420 nm)	5℃	10 mg	14170 μmol/g/h	[3]
MoS_2-tipped CdS nanorod	25 mg/mL PET	300 W Xe lamp (λ > 400 nm)	5℃	1.66 mg/mL	3900 μmol/g/h	[4]
CN_x∣Pt	25 mg/mL PET in 10 M KOH	1000 W Xe lamp (AM 1.5G)	25℃	1.6 mg/mL	81 μmol/g/h	[5]
CNx∣Ni_2P	25 mg/mL PET in 10 M KOH	AM 1.5G	25℃	1.6 mg/mL	83 μmol/g/h	[5]
H_2NCNx∣Ni_2P	25 mg/mL PET in 10 M KOH	1000 W Xe lamp (AM 1.5G)	25℃	1.6 mg/mL	110 μmol/g/h	[5]
CN-CNTs-NiMo	50 mg/mL PET in 5 M KOH	500 W Xe lamp	15℃	10 mg	90 μmol/g/h	[6]
High-entropy oxide (Pt/$TiZrHfTaNbO_{11}$)	50 mg PET in 3 ml of 10 M NaOH	300 W Xe lamp (18 kW/m^2)	25℃	50 mg	319 μmol/m/h	[7]
TiO_2/ Pt (8wt%)	50 mg PET Enzymatic pretreatment Leaf-compost cutinase	Solar light simulator (100 mW cm^{-2})	25℃	2 mg	518 μmol/g/h	[8]
Pt (3wt%) g-C_3N_4	200 mg/mL PET in 0.1 M NaOH	Visible light (λ > 400 nm)	Room temperature	50 mg	2000 μmol/g/h	[9]
0.6 wt% Cu-ZnS	25 mg/mL PET in 5 M KOH	300 W Xe lamp (λ > 400 nm)	25℃	20 mg	201.5 μmol/g/h	This work

References

1. Zhang, S.; Li, H.; Wang, L.; Liu, J.; Liang, G.; Davey, K.; Ran, J.; Qiao, S.-Z. Boosted Photoreforming of Plastic Waste via Defect-Rich NiPS3 Nanosheets. J. Am. Chem. Soc. 2023, 145, 6410–6419, https://doi.org/10.1021/jacs.2c13590.
2. Uekert, T.; Kuehnel, M.F.; Wakerley, D.W.; Reisner, E. Plastic waste as a feedstock for solar-driven H2 generation. Energy Environ. Sci. 2018, 11, 2853–2857, https://doi.org/10.1039/c8ee01408f.
3. Cao, B.; Wan, S.; Wang, Y.; Guo, H.; Ou, M.; Zhong, Q. Highly-efficient visible-light-driven photocatalytic H2 evolution integrated with microplastic degradation over MXene/ZnxCd1-xS photocatalyst. J. Colloid Interface Sci. 2022, 605, 311–319, https://doi.org/10.1016/j.jcis.2021.07.113.
4. Du, M.; Zhang, Y.; Kang, S.; Guo, X.; Ma, Y.; Xing, M.; Zhu, Y.; Chai, Y.; Qiu, B. Trash to Treasure: Photoreforming of Plastic Waste into Commodity Chemicals and Hydrogen over MoS2-Tipped CdS Nanorods. ACS Catal. 2022, 12, 12823–12832, https://doi.org/10.1021/acscatal.2c03605.

5. Liu, Y.-X.; Wang, H.-H.; Zhao, T.-J.; Zhang, B.; Su, H.; Xue, Z.-H.; Li, X.-H.; Chen, J.-S. Schottky Barrier Induced Coupled Interface of Electron-Rich N-Doped Carbon and Electron-Deficient Cu: In-Built Lewis Acid–Base Pairs for Highly Efficient CO2 Fixation. J. Am. Chem. Soc. 2018, 141, 38–41, https://doi.org/10.1021/jacs.8b08267.
6. Gong, X.; Tong, F.; Ma, F.; Zhang, Y.; Zhou, P.; Wang, Z.; Liu, Y.; Wang, P.; Cheng, H.; Dai, Y.; et al. Photoreforming of plastic waste poly (ethylene terephthalate) via in-situ derived CN-CNTs-NiMo hybrids. Appl. Catal. B: Environ. 2022, 307, https://doi.org/10.1016/j.apcatb.2022.121143.
7. Nguyen, T.T.; Edalati, K. Efficient photoreforming of plastic waste using a high-entropy oxide catalyst. J. Catal. 2024, 440, https://doi.org/10.1016/j.jcat.2024.115808.
8. Bhattacharjee, S.; Guo, C.; Lam, E.; Holstein, J.M.; Pereira, M.R.; Pichler, C.M.; Pornrungroj, C.; Rahaman, M.; Uekert, T.; Hollfelder, F.; et al. Chemoenzymatic Photoreforming: A Sustainable Approach for Solar Fuel Generation from Plastic Feedstocks. J. Am. Chem. Soc. 2023, 145, 20355–20364, https://doi.org/10.1021/jacs.3c05486.
9. Li, M.; Zhang, S. Tandem Chemical Depolymerization and Photoreforming of Waste PET Plastic to High-Value-Added Chemicals. ACS Catal. 2024, 14, 2949–2958, https://doi.org/10.1021/acscatal.3c05535.
